# A Perspective on Extracellular Vesicles Proteomics

**DOI:** 10.3389/fchem.2017.00102

**Published:** 2017-11-21

**Authors:** Livia Rosa-Fernandes, Victória Bombarda Rocha, Victor Corasolla Carregari, Andrea Urbani, Giuseppe Palmisano

**Affiliations:** ^1^GlycoProteomics Laboratory, Department of Parasitology, Institute of Biomedical Sciences, University of São Paulo, São Paulo, Brazil; ^2^Proteomic and Metabonomic Laboratory, Fondazione Santa Lucia, Rome, Italy; ^3^Institute of Biochemistry and Biochemical Clinic, Università Cattolica del Sacro Cuore, Rome, Italy

**Keywords:** extracellular vesicles, exosomes, proteomics, post-translational modification, mass spectrometry, bottom-up, top-down, crosslinking

## Abstract

Increasing attention has been given to secreted extracellular vesicles (EVs) in the past decades, especially in the portrayal of their molecular cargo and role as messengers in both homeostasis and pathophysiological conditions. This review presents the state-of-the-art proteomic technologies to identify and quantify EVs proteins along with their PTMs, interacting partners and structural details. The rapid growth of mass spectrometry-based analytical strategies for protein sequencing, PTMs and structural characterization has improved the level of molecular details that can be achieved from limited amount of EVs isolated from different biological sources. Here we will provide a perspective view on the achievements and challenges on EVs proteome characterization using mass spectrometry. A detailed bioinformatics approach will help us to picture the molecular fingerprint of EVs and understand better their pathophysiological function.

## Introduction

In the past decade, secreted extracellular vesicles (EVs) have been recognized as important molecular messengers for intercellular communication in prokaryotes and eukaryotes (Yáñez-Mó et al., 2015). Furthermore, EVs have been involved in cellular homeostasis compensating for the stress conditions and provided a novel physiological role in maintenance of cellular integrity and organismal homeostasis (Takeuchi et al., 2015; Desdín-Micó and Mittelbrunn, 2016). Their involvement in different pathophysiological processes has been highlighted in several excellent reviews (Schorey and Bhatnagar, 2008; Mathivanan et al., 2010) and will not be covered here. The term EV categorizes different vesicles based on their biogenesis or release pathway, such as exosomes (30–100 nm in diameter), ectosomes, or shedding microparticles/microvesicles (100–1,000 nm; Heijnen et al., 1999), apoptotic blebs (50 nm−2 μm), oncosomes (1–10 μm), and other EV subsets, as reviewed by the International Society of Extracellular Vesicles (ISEV, www.isev.org). Through this review, we will predominantly use the term EVs except in studies which investigated a specific enriched vesicle population.

The composition of extracellular vesicles is not random, but each EVs cargo delivers specific molecular messages. Indeed, these nanosized membrane vesicles transmit EV-mediated signals by proteins, lipids, nucleic acids and sugars, and the unique molecular pattern of this package dictate the type of extracellular signal to be transmitted to recipient cells. The protein cargo of EVs are cell- and disease-type related and confer particular features to the extracellular vesicles influencing their biological properties (Pegtel et al., 2014; Kalra et al., 2016; Tkach and Thery, 2016).

Protein components of extracellular vesicles derived from different cell types and biofluids have been cataloged using proteomic technologies, western blotting and fluorescent-activated cell sorting (Pisitkun et al., 2004; Miguet et al., 2006; Lee et al., 2008). In particular, mass spectrometry (MS)-based proteomic analysis has boosted our knowledge about the protein content of EVs. The past decades have seen proteomics technologies dominating the scenario of protein identification and quantification (Mallick and Kuster, 2010; Cox and Mann, 2011). Especially, bottom up mass spectrometry-based proteomics has been used worldwide as the strategy of choice. In this approach proteins are extracted from a biological source, digested into peptides which are subsequently separated by 1D or 2D gel electrophoresis (Gel-based) or liquid chromatography (Gel-free) and analyzed by mass spectrometry. Peptide ions are fragmented in the gas phase and their sequence and PTMs can be deduced. Moreover, quantitative information on peptides and proteins can be deduced. Protein quantification can be achieved using different strategies depending on the aim (Domon and Aebersold, 2010). In particular, shotgun proteomics approach allows a discovery-driven protein identification and quantification in which peptide ions are measured and heuristically selected for fragmentation using a data-dependent mode (Wolters et al., 2001). In targeted proteomics, only predetermined peptide ions are selected for fragmentation allowing a hypothesis-driven protein detection and quantification. Several acquisition methods have been implemented for targeted proteomics such as selected reaction monitoring (SRM; Lange et al., 2008), pseudo selected reaction monitoring (pSRM; Sherrod et al., 2012), and parallel reaction monitoring (PRM; Gallien et al., 2012; Peterson et al., 2012). An SRM experiment is usually acquired on triple quadrupole instruments and involves the selection of specific precursor/fragment ion pairs, named transitions, belonging to the target peptide that is used as a surrogate for the protein of interest. On the contrary, pSRM and PRM approaches monitor all fragment ions for each selected peptide precursors. Because of the simultaneous monitoring of all fragment ions, pSRM and PRM do not need the selection of transitions. pSRM is usually performed in LTQ or LTQ-Orbitrap instruments while PRM is usually performed in hybrid quadrupole-Orbitrap or quadrupole-TOF instruments. A direct comparison between SRM and PRM using 35 isotopically labeled peptides spiked in urine as the biological matrix, showed that PRM has high selectivity, due to the high resolution in the MS/MS stage while SRM has, in some cases, higher sensitivity associated to lower limit of quantification (Gallien et al., 2013). Rosein et al. evaluated the ability of PRM and SRM approaches to quantify proteins in high density lipoproteins. This study reported similar performances in terms of dynamic range, precision, and linearity between PRM and SRM methods (Ronsein et al., 2015).

Another approach is the data-independent acquisition in which no precursor ions selection occurs and all precursors are fragmented (Venable et al., 2004; Gillet et al., 2012; Egertson et al., 2013). The resulting MS/MS spectra are commonly searched using spectral libraries (Gillet et al., 2012) or novel computational frameworks (Tsou et al., 2015). Diverse proteomics approaches have been used in EVs characterization and several reviews have been published in that matter (Choi et al., 2015; Kreimer et al., 2015; Abramowicz et al., 2016). Currently (June 2017) there are 47 available datasets open to the scientific community in the proteomexchage (http://proteomecentral.proteomexchange.org) repository. The first submissions were released in 2014 and since then the number of available datasets have been doubled providing raw data from different species: *Homo sapiens, Bos Taurus, Equus caballus, Mus musculus, Saccharomyces cerevisae and pombe, Fasciola hepatica, Mycobacterium tuberculosis*.

In this review, we will focus on several aspects of EVs preparation that should be considered when combined to mass spectrometry-based protein analysis, along with novel proteomic tools used to better understand the dynamic features of EVs proteome, for example post-translational modification and protein-protein interaction.

## EVs isolation and proteomics

The plurality of isolation methods has been an important limitation to characterize a well define EV population. Indeed, cells secrete a heterogeneous population of EVs which differ in size and cargo. A proper control of the EVs population can help in understanding the biology of specific EVs and identifying disease biomarkers (Simpson et al., 2009; Properzi et al., 2013; Sandfeld-Paulsen et al., 2016; Thind and Wilson, 2016).

In this section, we will analyse the current EVs isolations methods and their advantages and disadvantages in the light of a mass spectrometry-based proteomic analysis of EVs proteins. Moreover, a specific focus on EVs protein post-translational modifications, along with their potential for disease biomarker and therapeutic intervention, will be provided in section Advanced Mass Spectrometry-Based Proteomics Strategies for EVs Protein Analysis.

### EVs isolation methods coupled to proteomics strategies

EVs isolation methods have been refined along the years. To date, there is no EVs isolation protocol that allows the recovery of a pure EVs subpopulation and the majority of the available protocols have profound influence on the omics results applied post-isolation (Tauro et al., 2012). Due to that, proteomic profiles of EVs have been highly dependent on the isolation protocol. Therefore, it is important to include in EVs studies all the detailed information for definition of extracellular vesicles and their function (Witwer et al., 2013; Lötvall et al., 2014) according to the International Society for Extracellular Vesicles guidelines.

Evaluation of morphological and protein markers for a EVs isolation is not sufficient. Indeed, non-exosomal contaminants should always be assessed and better protein and/or lipid markers should be defined. Appraisal of a proper protocol for EVs isolation depends on the type of biological sample and molecular characterization. Indeed, isolating EVs from conditioned medium of cell culture presents a different challenge compared to blood or urine. Moreover, profiling mRNA, proteins or lipids require different level of purity due to the specificity of the technique and potential contaminants. This review focuses on EVs proteomic and isolation methods presented here will be evaluated based on their biological source and compatibility toward a mass spectrometry-based proteomic analysis. Indeed, isolation from cell conditioned medium is more challenging compared to plasma or other biofluids due to the high dynamic range and potential protein contaminants. Differential ultracentrifugation has been used in the early EVs preparations (Raposo et al., 1996). This method is based on several steps of centrifugation with a final ultracentrifugation step to isolate small extracellular vesicles. The resulting pellet containing EVs can be solubilized in PBS and directly subjected to in-solution digestion or separated by 1D or 2D gel electrophoresis before LC-MS analysis (Welton et al., 2010). Protein aggregates could be co-isolated reducing the sample purity. Considering that, the exosomal pellet can be further purified with sucrose or iodixanol density gradient centrifugation. These gradients can be continuous or discontinuous. After density gradient centrifugation the EVs are present in large volumes. Due to that, another step such as ultracentrifugation, precipitation, membrane filtration, or size exclusion chromatography is needed to have a purified exosomal pellet. Exosomes isolated from plasma are more likely subjected to the “contamination” of soluble plasma proteins and/or protein complexes and aggregates. In light of that, exosome preparations should be verified using negative markers such as albumin and apolipoproteins. However, it should be noted that some serum proteins might be associated to exosomes such as complement components (Papp et al., 2008) or IgG (Ramirez-Alvarado et al., 2012).

Another method for EVs isolation is polymer-based precipitation. This method is based on mixing the sample with a polymer solution which creates, at specific salt conditions and temperature, a polymer network allowing the isolation of EVs by low-speed centrifugation. Beside commercial kits available such as ExoQuick^TM^ (System Biosciences) and Total Exosome Isolation kit (Thermo Fisher Scientific), polyethylene glycol (PEG) is commonly used (Rider et al., 2016). After centrifugation EVs are resuspended in PBS for further analyses. However, EVs isolated by the precipitation method contain excess of salts and polymer not compatible with mass spectrometry. Due to that, protein precipitation, 1D gel electrophoresis and membrane filtration can be used to clean up the sample before LC-MS analysis. Recently a novel PRotein Organic Solvent PRecipitation (PROSPR) method was reported to isolate exosomes from 500 to 1,000 μl of blood (Gallart-Palau et al., 2015). The method is rapid and inexpensive based on the direct mixing of plasma with cold acetone in a 1:4 volume. After centrifugal removal of blood proteins, exosomes present in the supernatant can be isolated by membrane filtration or solvent evaporation. The method was compared to differential centrifugation showing not only the characteristic EV protein markers such as CD9, CD63, Alix, and CD81 but also more pure EVs preparation. After sample evaporation, the pellet was resuspended in urea buffer for in-solution digestion and LC-MS analysis. Proteomic analysis of PROSPR-isolated EVs allowed the identification of 1539 proteins (Gallart-Palau et al., 2015). It should be noted that the precipitation method results in higher protein amount compared to the ultracentrifugation method.

Another method for EVs isolation is membrane filtration which uses membranes with 100 kDa cut-off to remove salts, small molecules and soluble proteins and retain exosomes. This method suffers for sample loss due to unspecific membrane absorption and the possibility of retention of high molecular weight proteins or protein aggregates. It can be applied to samples present in large volumes such as urine (Cheruvanky et al., 2007) and cell culture media (Lobb et al., 2015). Size exclusion chromatography (SEC) utilizes the sepharose gel filtration medium to separate vesicles from soluble proteins and small molecules. In particular, small molecules enter the pores of the chromatographic medium while vesicles are not retained and eluted earlier. Recently, plasma EVs were isolated using the PEG, PROSPR, and size exclusion chromatography (SEC) method. It was shown that SEC gave the highest pure EVs due to the removal of the highly abundant soluble plasma proteins based on Cryo-EM analysis and the low protein content. Moreover, only SEC allowed the detection of the EV-markers CD9, CD63, and CD81, LGALS3BP and CD5L, suggesting a putative interference of the precipitating agents in the structure/composition of the EVs (Gámez-Valero et al., 2016). Furthermore, PEG and PROSPR-based EV isolation resulted in reduced cell viability *in vitro* (Gámez-Valero et al., 2016).

Affinity-based EVs isolation employ antibodies against epitopes of EVs markers such as CD63, CD9, CD81, annexin, or EpCAM. The immunoaffinity methods are the most specific since they target one or more surface exosomal proteins. However, this specificity can be a drawback for capturing the whole EVs population. Immuno-affinity isolation of EVs has been coupled to LC-MS analysis showing its feasibility to proteomic strategies (Tauro et al., 2013).

Recently a novel method based on affinity capture using a synthetic peptide with high affinity toward heat shock proteins was developed (Ghosh et al., 2014). The method proved to be efficient in aggregating HSP-decorated EVs and their morphological and protein content were similar to EVs isolated by differential centrifugation.

Understanding the purity of a EVs isolation protocol is important to derive meaningful results. Despite the use of electron microscopy and GRP94, cytochrome C, GM130, calnexin, or gp96 as exclusion biomarkers, Webber and Clayton proposed that 3 × 10^10^ particles would correspond to 1 μg of proteins for high vesicular purity, while ratios of 2 × 10^9^-2 × 10^10^ P/μg would indicate low purity (Webber and Clayton, 2013).

Some of these protocols have been extensively compared in several articles. Tauro et al. performed a comprehensive evaluation of the ultracentrifugation, density gradient separation, and immunoaffinity capture methods for the isolation of exosomes from LIM1863 colorectal cancer cell. Based on the number of MS/MS spectra of exosomal protein markers such as Alix, TSG101, CD9, and CD81, they concluded that the immunoaffinity capture method was most effective (Tauro et al., 2012). It should be noted that exosome affinity purification from biofluids could suffer from co-isolating contaminants due to unspecific binding to the resin/antibody.

Another study compared differential and density gradient centrifugation with commercially available precipitation kits to isolate exosomes from breast cancer cell conditioned medium (Van Deun et al., 2014). Optiprep^TM^ density gradient ultracentrifugation had the highest level of CD63 and other exosomal markers compared to precipitation protocols although the number of particles and protein yield was two-fold less. Moreover, using immunoelectron microscopy with anti-CD63 antibody the authors showed that density gradient ultracentrifugation displayed the most heterogenous exosomal population. Another study compared isolation protocols for exosomes derived from plasma. The OptiPrep^TM^ density gradient method allowed pure exosomes without co-isolating plasma proteins such as albumin and apolipoprotein (Kalra et al., 2013).

Recently, different isolation methods of exosomes derived from plasma and urine were evaluated in the context of a hospital setting (Sáenz-Cuesta et al., 2015). Several isolation protocols are difficult to implement in a hospital setting since they are time-consuming or require specific infrastructure. This study defined a medium-speed differential centrifugation as the best-suited EVs isolation protocol in a hospital setting (Sáenz-Cuesta et al., 2015). More studies are needed to evaluate the applicability of exosomes in translational diagnostics and therapeutics (Lener et al., 2015).

#### The isolation of EVs from cultured cell lines and biofluids and their effect on the EVs proteome

As cell culture experiments often involve the presence of fetal bovine serum (FBS), it is possible that vesicles present in the FBS can influence experimental results. Indeed, it has been suggested that FBS-derived EVs may influence results in cell biology, such as growth of breast cancer cell lines (Ochieng et al., 2009). Also, FBS-derived EVs are a major cause of concern as these vesicles could contaminate EVs derived from cell cultures. Therefore, EVs are often removed from the FBS by the use of ultracentrifugation-based depletion protocol (Théry et al., 2006). Recently it was shown that FBS-derived RNA species are co-isolated with cell-derived extracellular RNA causing an important confounding factor in RNA sequencing experiments such as FBS-specific miRNA: miR-122, miR-451a, and miR-1246 annotated as cell derived EVs (Wei et al., 2016). A way of removing “contaminating” serum proteins from the EVs preparation is through serum starvation. This procedure avoids contamination with FBS EVs and improves the identification of *bona fide* exosomal biomolecules. However, serum starvation presents several caveats and below we will analyse them and provide possible solutions.

Serum starvation is known to induce profound molecular changes in the biochemical pathways landscape. Indeed, serum starvation can activate cell cycle arrest in the G0/G1 phase and synchronization along with induced-stress responses which alters the metabolic flux of nutrients. These effects vary across different cell types and experimental conditions. Due to that, serum starvation clearly represents a major event which triggers a plethora of divergent responses and has therefore great potential to interfere with the experimental results and affect subsequent conclusions (Eichelbaum et al., 2012). Li et al. showed that exosomes derived from neuroblastoma cell lines cultured in serum-free medium differed in amount and protein expression compared to exosomes-depleted serum conditions (Li et al., 2015). These results highlight the importance of intracellular pathways in modulating the quantity and content of EVs. Using 1% of bovine serum albumin has been proposed for some cell lines but the results should be evaluated based on each biological conditions (Théry et al., 2006).

Nowadays several studies are using exosome-free serum, obtained by ultracentrifugation, to remove bovine serum exosomes and avoid changes in the cell biology. FBS ultracentrifugation for 18-h has been shown to remove 95% of RNA-containing FBS EVs (Shelke et al., 2014). It should be noted that, for protein identification purposes, the presence of FBS-derived EVs is detrimental since high sensitive mass spectrometers can detect and erroneously assign them.

Another important issue in EVs isolation is the high abundance of specific proteins in biofluids. These proteins can unspecifically bind to EVs and reduce the MS sensitivity. Indeed, it has been noticed that high abundance proteins hinder a deeper identification of the EVs proteome. In urine, the high abundance 68 kDa protein uromodulin co-fractionate with exosomes, being a contaminant, which limits the MS sensitivity and, so on, the number of identified proteins. Thermochemical (Pisitkun et al., 2004) and centrifugal (Hogan et al., 2009) methods have been proposed to remove uromodulin for exosome preparation and improve the protein identification; however these methods influence the sample quality altering the protein contents of EVs. As another option, MS-based filtering was implemented using the m/z exclusion list. In this strategy, high abundance m/z ions of uromodulin peptides were deliberately excluded for fragmentation (Hiemstra et al., 2011) resulting in an increase in identification of 77%. It should be noted that in these experiments, the m/z window is important since it can cause the co-isolation of other peptides reducing the number of identifications. Street et al. evaluated the impact on CSF exosomal proteome characterization by removing immunoglobulins. Indeed, CSF samples were immunoglobulin depleted by protein G agarose beads incubation after ultracentrifugation. Proteins were extracted using chloroform/methanol precipitation, separated by PS-DVB column and sequenced using an FT-ICR instrument in top-down approach (Street et al., 2012). An elegant approach to enrich, identify and quantify *bona fide* secreted proteins was developed using the incorporation of non-canonical amino acids functionalized with bio-orthogonal groups and combined with pulsed stable isotope labeling with amino acids in cell culture. After the incorporation of these amino acids, secreted proteins were fished out using click chemistry reactions before mass spectrometry-based identification (Eichelbaum et al., 2012). This strategy was applied to immortalized and primary cell cultures. In this respect, the secretome of primary hepatocytes was markedly different from hepatoma cell lines. This conclusion opens a debate on the nature of secreted EVs isolated from primary or immortalized cell lines and more studies are needed to investigate these discrepancies. It should be noted that exosome have been isolated also *in vivo* using fresh and frozen brain tissues. Indeed, a specific protocol based on gentle tissue lysis using mild papain treatment followed by differential centrifugation in a sucrose gradient. The full length amyloid β precursor protein and its carboxyl-terminal fragments were identified in exosomes isolated from mouse and human brain tissues showing the importance of extracellular vesicles in releasing neurotoxic proteins (Perez-Gonzalez et al., 2012). Another study reported the isolation of exosomes from human and mouse brain using the protein organic solvent precipitation (PROSP) after mild tissue homogenization using metallic beads at low speed (Gallart-Palau et al., 2016).

Nowadays, state-of-the-art proteomic technologies allows the identification of thousands proteins from minute amount of sample, for example FACS-sorted (Di Palma et al., 2011; Maurer et al., 2013), laser captured microdissected (LCM), formalin fixed cells (Waanders et al., 2009), or even fine-needle aspiration biopsy (Giusti et al., 2008). Moreover, optimized sample preparation techniques with minimal sample can further improve protein identification (Kulak et al., 2014). Every EVs isolation protocol needs to be adjusted on the need for functional assays. The different proteomic strategies available nowadays do not require biologically functional vesicles and several isolation methods can be used prior to EVs biomolecular characterization.

There is no optimal EVs isolation method, which guarantees a pure and homogeneous population. Due to that, it is challenging to define specific protein markers for a specific EV subpopulation. In the next paragraph, we will evaluate the current protein markers reported for EVs classification.

### EVs protein markers

The proteome content of EVs is dependent not only on the parental cell type and conditions in which the EVs are secreted, but is also based on the type of EVs. The isolation of a pure population of EVs is not achievable due to overlapping physicochemical properties. Proteins enriched in EVs are often used as markers to demonstrate the purity of a EVs preparation. Indeed, tetraspanins (CD9, CD63, CD81, and CD82), 14-3-3 proteins, major histocompatibility complex (MHC), heat shock proteins, Tsg101 and the Endosomal Sorting Complex Required for Transport (ESCRT-3) binding protein Alix are consider as “specific” exosomes markers. However, these proteins have also been identified in apoptotic bodies and microvesicles (Crescitelli et al., 2013; Tauro et al., 2013).

**Figure 1:**
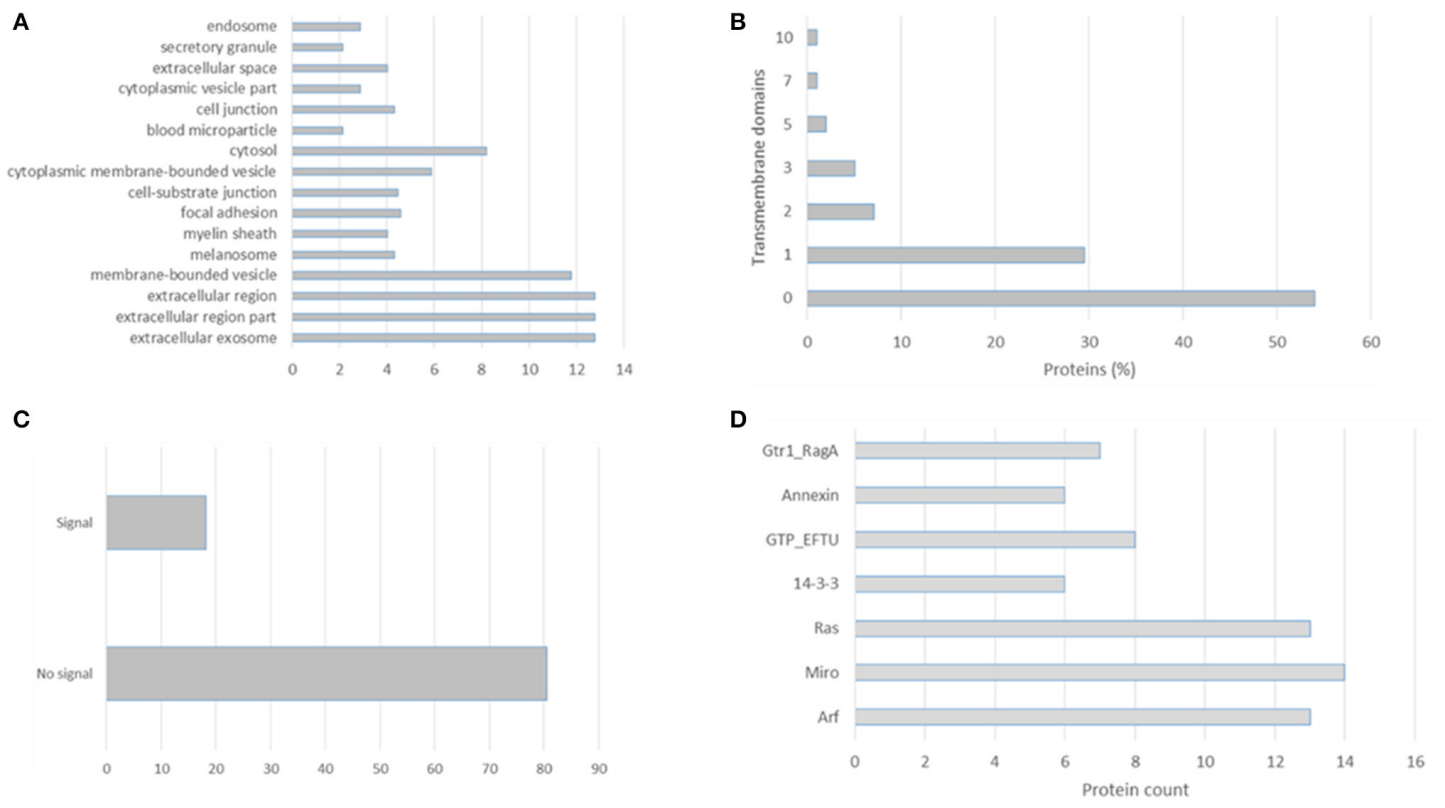
Gene Ontology analysis of the top 100 proteins identified in exosomal preparations as reported in the Exocarta database. **(A)** Cellular components, **(B)** transmembrane domains, **(C)** signal peptide, and **(D)** enriched domains were analyzed using the ProteinCenter Software (Thermo Fisher).

EVpedia (evpedia.info/; Kim et al., 2015), Exocarta (www.exocarta.org/; Mathivanan and Simpson, 2009), Vesiclepedia (www.microvesicles.org/; Kalra et al., 2012), and Plasma Proteome Database (http://plasmaproteomedatabase.org/; Nanjappa et al., 2014) are the main four databases on extracellular vesicles biomolecules. These curated databases provide a compendium of proteins, lipids, and RNA which have been identified in several EVs preparations. Their importance in cataloging allow data mining by different researchers and further comparisons. In this review we distilled the 100 most identified proteins in the Exocarta database (Supplementary Table 1), the top 100 identified proteins in the EVpedia database (Supplementary Table 2) and the extracellular vesicles isolated from plasma (Supplementary Table 3). Firstly, we analyzed the cellular components, transmembrane domains, signal peptides and PFAM domains of the top 100 proteins identified in exosomal preparation according to the Exocarta database, Supplementary Table 4. These proteins were localized to extracellular region, endosome, and exosomes but also cytoplasm (Figure 1A). More than 40% of these proteins had at least one transmembrane domain confirming the 28–34% estimation of membrane and membrane-associated proteins in biofluids and conditioned media-derived EVs (Raimondo et al., 2011), Figure 1B. The non-canonical secretion of exosomal proteins can be visualized in Figure 1C in which 80% of the top 100 most identified exosomal proteins had no signal peptide. Moreover, an enrichment of the PFAM domains revealed the ADP Ribosylation Factors family (ARFs) domain which is related to vesicle biogenesis and intracellular trafficking. The other domains enriched in this exosomal protein dataset were the Miro and Ras domains which are related to GTPases involved in vesicles biogenesis and the 14-3-3 protein family related to cellular signaling and the GTP-binding elongation factor family related to elongation factors (Figure 1D).

**Figure 2:**
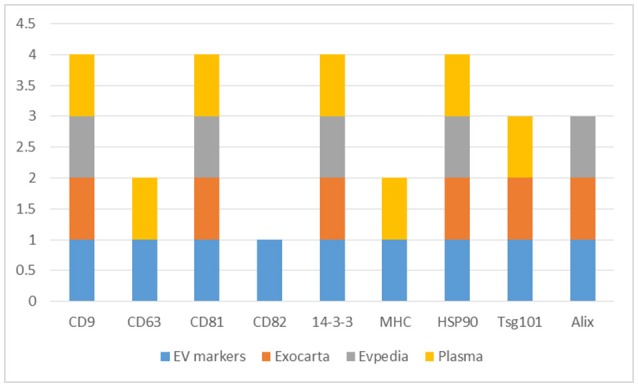
Distribution of the EVs protein markers (CD9, CD63, CD81, CD82, 14-3-3, MHC, HSP90, Tsg101, and Alix) in the Exocarta, EVpedia, and Plasma Proteome Database Extracellular Vesicles.

**Figure 3:**
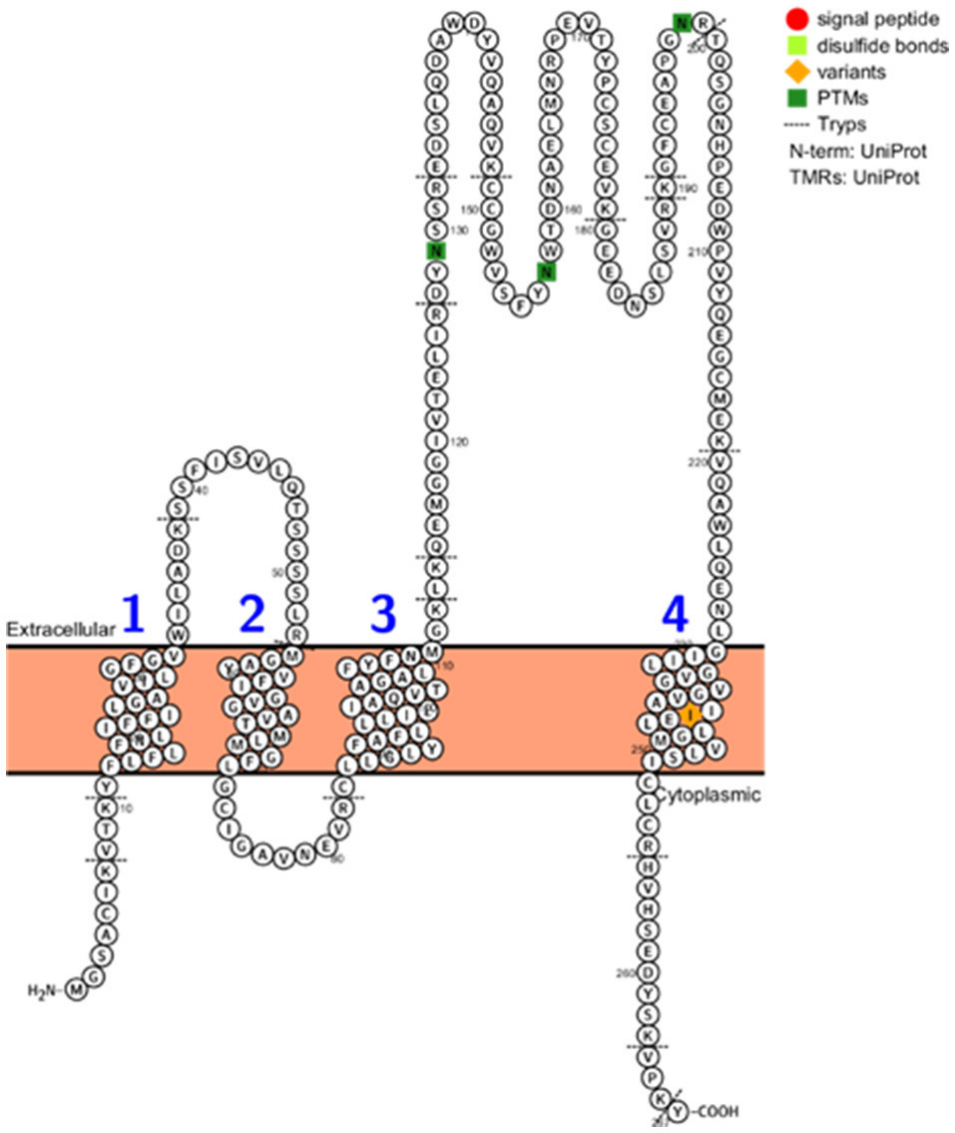
CD82 protein topology, PTMs, variants, disulphide bonds, signal peptide, and tryptic digest sites visualized using the Protter web tool (http://wlab.ethz.ch/protter/start/).

The CD9, CD63, CD81, and CD82, 14-3-3 proteins, major histocompatibility complex (MHC), HSP90, Tsg101, and Alix proteins were searched in the three datasets, Figure 2 and Supplementary Table 5. The CD9, CD81, 14-3-3, and HSP90 proteins were identified in all three datasets while Tsg101 and Alix were identified in two datasets. The CD63 and MHC proteins were identified only in the EVs isolated from plasma and interestingly the CD82 protein was not present in any of the datasets. CD82 is a membrane protein with four transmembrane domains and the N- and C-termini located into the cytoplasmic part. This protein had three N-linked glycosylation sites located on the extracellular part. One reason for the missed identification of CD82 in the three datasets could be due to the generation of tryptic peptides which are not suitable to MS analysis based on their size, Figure 3.

Searching for EV-subgroups protein markers has been a priority to isolate specific EVs subpopulations. Recently a comprehensive quantitative proteomic analysis of EVs isolated from human primary dendritic cells was performed. EVs were firstly separated by differential centrifugation and the common exosome markers such as MHC, flotillin, and HSP70 were identified in all EVs independently of their sedimentation speed. Subsequently, EVs were isolated by floatation into iodixanol gradients or by immuno-isolation and the different fractions were compared using quantitative label-free proteomic strategy. It was found that tumor susceptibility 101 (TSG101), syntenin-1, EHD4, Annexin XI, and ADAM10 are better protein markers for small EVs than the classical exosomal protein markers previously used. These protein markers were also verified in small EVs isolated from other cell lines. Interestingly, TSG101, synthenin-1, EHD4, and annexin A6 are present within the 100 mostly identified exosomal proteins according to the Exocarta database. Moreover, this study showed the possibility of immunoaffinity-based isolation of subpopulations of small EVs (Kowal et al., 2016).

We believe that a single isolation method might not unravel the total and specific proteomic content of EVs. Due to that, the EV research community could indicate a combination of more than one method, when possible, to describe the proteome content of a particular EV population.

## Advanced mass spectrometry-based proteomics strategies for EVs protein analysis

### Post-translational modifications in EVs: an underexplored world

Current high resolution, accuracy, and sensitive mass spectrometry-based analyses allow the identification and quantification of thousands of EVs proteins [P. Tomlinson (2015)117, A. Sinha (2014)116] suggesting that the technology is ready for large-scale portrait of the “EVome.” However, PTMs and conformational changes can add other layers on protein dynamics regulation. Post-translational modifications can effect protein structure and function by changing its interactors and enzymatic activity (Jensen, 2006). At this time, more than 450 PTMs are annotated in the Uniprot database (Venne et al., 2014). More than 500 kinases, almost 200 phosphatases and more than 500 proteases highlight the role of PTMs in human cells, tissues, and biofluids with a total of more than 5% of the human genome coding for regulatory enzymes involved in PTMs assembly (2012). Elucidating the PTM code in EVs could allow the identification of PTM-specific proteoforms secreted in EVs and used to modulate the microenvironment. The post-translational modification in EVs might help in understanding the sorting mechanisms and identify novel PTM-specific moonlight functions of proteins secreted in EVs. The following paragraph will discuss the technological approaches and biological implications of PTMs characterization in EVs. In particular, we will comment on the recent advancements on EVs protein phosphorylation, glycosylation and ubiquitylation. Recently, detailed reviews on different post-translational modifications in EVs and their role in biogenesis have been reported (Moreno-Gonzalo et al., 2014; Szabó-Taylor et al., 2015). As discussed above, the state-of-the-art proteomic approaches are able to identify the proteome expressed by an organism. However, for PTM analysis the scenario is different. In order to identify specific PTMs, the modification should be stable during sample preparation and LC-MS analysis. Indeed, phosphatases or GlcNAcase inhibitors are commonly added during cell or tissue lysis to prevent phosphorylation or GlcNAc loss, respectively (Pan et al., 2008; Ahmed and Gardiner, 2011). The modification should add a mass shift compared to the unmodified peptide in order to be detected by mass spectrometry (Jung et al., 2013; Kim et al., 2016). A further complication are isobaric or nearly isobaric modifications which need specific chemical (Xu and Jaffrey, 2013), enzymatic or LC-MS strategies (Zhang et al., 2006; Jung et al., 2013; Webber and Clayton, 2013) to be differentiated (Kim et al., 2016). Moreover, modified peptides are present in low abundance and sub-stoichiometric ratio compared to unmodified peptides, are poorly ionized in MS and underrepresented in a typical proteolytic digested proteome. Due to that, specific enrichment methods have been implemented for each PTM such as metal affinity chromatography [IMAC (Ficarro et al., 2002) and TiO_2_ (Pinkse et al., 2004; Larsen et al., 2005)] for phosphorylation, lectins (Lee et al., 2010), and HILIC (Mysling et al., 2010) for glycosylation and antibodies for ubiquitylation (Kim et al., 2011). The MS/MS behavior of modified peptides is still the subject of intensive research to interpret the fragmentation spectra of modified peptides. Different fragmentation techniques such as ECD/ETD have been used to fragment labile PTMs such as phosphorylation (Chi et al., 2007) and glycosylation (Mechref, 2012). These techniques are important to correctly localize the modification site and functionally study them in a site-specific manner. Moreover, the correct interpretation of MS/MS spectra has allowed the development of localization algorithms helping the computational analysis of large scale mass spectrometry-based PTM analyses (Chalkley and Clauser, 2012). Relative quantification has been achieved using metabolic, such as SILAC, and chemical labeling and label-free approaches. Phosphorylation (Olsen et al., 2010; Wu et al., 2011), acetylation (Baeza et al., 2014; Weinert et al., 2015), and ubiquitylation (Udeshi et al., 2012) stoichiometry has been investigated using chemical and enzymatic methods combined with different MS strategies. The PTM catalog of a biological system has not yet been reached and more studies are needed to expand our knowledge in different biological systems such as EVs.

#### Phosphorylation in EVs

Protein phosphorylation is one of the most common reversible PTM, catalyzed by kinases and removed by phosphatases which regulates several signaling events intra and extracellularly. The most studied phosphorylation is O-phosphorylation on serine, threonine and tyrosine with 1,800:200:1 ratio. Comprehensive reviews on sample preparation and LC-MS analysis of protein phosphorylation have been reported elsewhere (Steen et al., 2006; Dephoure et al., 2013; Engholm-Keller and Larsen, 2013; Solari et al., 2015). Protein phosphorylation in EVs has been studied on single proteins. Indeed, protein phosphorylation has been involved in EVs biogenesis through phosphorylation of myosin light chain mediated by the ERK pathway through the ARF6 protein (Muralidharan-Chari et al., 2009). Moreover, phosphorylated and inactive MET was found to be transferred to exosomes secreted from advanced stage melanoma (Peinado et al., 2012). The aberrantly phosphorylated protein tau was identified in exosomes, suggesting a horizontal transfer of this proteoform in Alzheimer's disease patients (Saman et al., 2012). Recently, it was shown that active KRAS phosphorylated Ago2 through the MEK-ERK signaling pathways, inhibiting its association and sorting with exosomes along with specific miRNA (McKenzie et al., 2016). In exosomes isolated from glioma cells, crystalline alphaB (cryAB), a molecular chaperone with anti-apoptotic activity, was identified unphosphorylated compared to the extensive phosphorylation in the cytoplasmic form. Site-directed mutagenesis with phosphomimic amino acids interfered with the exosomal loading of cryAB. Moreover, abolishing the O-GlcNAc site on cryAB abolished its exosomal load (Kore and Abraham, 2016). Mass spectrometry-based phosphoproteomics approaches were applied in few studies while the above-mentioned ones used immunodetection techniques to address single protein phosphorylation. An initial study looked at exosomes isolated from NT1 insulinoma cell lines using differential centrifugation. The proteome profile was performed using a GeLC-MS approach with a nanoLC-Q-TOF acquisition allowing the identification of 270 proteins with at least two peptides. Moreover, PTMs were identified using a combination of a selectively excluded mass screening analysis (SEMSA) of unmodified peptides and MOD^i^ algorithm. Phosphorylation of HSP90-beta was identified (Lee et al., 2009). A phosphoproteomic workflow was applied to study exosomes isolated from urine. Differential centrifugation was combined with DTT reduction, since the abundant uromodulin protein form aggregates through disulphide linkages. After a GeLC-MS approach, 1132 proteins were identified. Moreover, titanium dioxide enrichment and data-dependent neutral loss scanning allowed the identification of 14 phosphoproteins that were further confirmed by immunoblotting (Gonzales et al., 2009). Transfer of kinases and phosphatases through EVs has been also reported (Putz et al., 2012; Fraser et al., 2013; Koumangoye and Delpire, 2016). Secreting aberrantly phosphorylated proteins and kinases/phosphatases in EVs can interfere with the signaling network of recipient cells having implications in diseased state. One example of phosphatase transfer through exosomes focused on the phosphatase and tensin homolog deleted on chromosome 10 (PTEN), a tumor suppressor protein. Putz U. et al. showed that PTEN is delivered through exosomes to recipient cells modulating their intracellular signaling pathways through reduced phosphorylation of Akt and reduced cellular proliferation (Putz et al., 2012). Montermini et al. identified in EVs phosphorylated epidermal growth factor receptor (EGFR) and other receptor tyrosine kinase a and showed that treatment of epidermal, breast, pancreatic, prostate, colon cancer cells with second generation EGFR kinase inhibitors (EKIs) modulated the EVs phosphoproteome and genomic-DNA content (Montermini et al., 2015). Taken together, these data add a predictive value to the EVs phosphoproteome cargo. Recently, a phosphoproteomic approach was applied to microvesicles and exosomes isolated from plasma of breast cancer patients (*n* = 18) and healthy subjects (*n* = 6). In total, 9,225 and 1,014 unique phosphopeptides were identified with 156 and 271 phosphosites significantly regulated in the microvesicles and exosome fractions, respectively. Three potential phosphopeptide markers belonging to the RALGAPA2, PRKG1, and TJP2 proteins were validated by PRM and showed to be significantly different between patients with breast cancer compared with healthy subjects (Chen et al., 2017).

#### Glycosylation in EVs

Protein glycosylation is one of most widespread co- or post-translational modification, which involves the covalent linkage of a glycan moiety to a protein by many elaborate biosynthetic routes involving glycosyltransferases and glycosidases located mainly in the ER and Golgi. Two of the most common forms of protein glycosylation are N- and O-linked glycoprotein. N-linked proteins have the glycan attached to asparagine within the motif Asn-X-Ser/Thr (where X≠P). In O-linked glycoproteins the glycan moiety is attached to serine or threonine without a unique motif. Membrane and secreted proteins are glycosylated, which alters their physicochemical and biological properties. Undeniably, protein glycosylation plays an important role in physiopathology of several diseases, including diabetes, cancer, cardiovascular, and neurodegenerative diseases (Moremen et al., 2012). Comprehensive reviews on sample preparation and LC-MS analysis of protein glycosylation have been reported elsewhere (Wells et al., 2001; Mariño et al., 2010; Moremen et al., 2012; Palmisano et al., 2013). One of the first reports of protein glycosylation in EVs used lectin microarray to profile the glycosylation of HIV-1 virion and microvesicles isolated from T-cells. HIV-1 and microvesicles shared common enriched glycan epitopes such as high mannose, complex N-linked glycans, N-acetyllactosamine, sialic acid, and fucosylated epitopes while were depleted in blood group antigen A/B compared to the parental cell line membrane glycoproteins (Krishnamoorthy et al., 2009). These data suggested a shared and glycan-dependent protein sorting mechanisms for HIV and microvesicles. The role of protein glycosylation in EVs protein sorting was shown for the EWI-2 protein. Indeed, inhibition of complex N-glycan formation inhibited EWI-2 sorting into EVs (Liang et al., 2014). The same lectin microarray platform was used to map the glycome of EVs isolated from different cell lines and human breast milk, showing a conserved glycomic profile (Batista et al., 2011). Likewise, lectin microarray and flow cytometry was used to analyse the glycome of exosomes isolated from urine of patients with polycystic kidney disease (Gerlach et al., 2013). In another approach, glycans of exosomes isolated from ovarian carcinoma SKOV3 cells were released by PNGase F, labeled with 2-AB and analyzed by MALDI-TOF/TOF or separated by normal and reversed phase chromatography before fluorescent and mass spectrometric detection. High mannose and di-, tri- ad tetra-antennary sialylated glycan structures were identified (Escrevente et al., 2013). Furthermore, the glycosylation of the galectin-3 binding protein (LGALS3BP), strongly enriched in exosomes isolated from OVMz ovarian cancer cells, revealed a prevalence of complex sialylated glycan structures (Gomes et al., 2015). The presence of specific glycan structures on LGALS3BP could modulate the interaction between exosomes and recipient cells influencing their delivery and uptake.

These studies focused on the glycan part, disregarding the site-specific micro and macro-heterogeneity. Recently, intact glycoproteomic approaches have been developed and optimized in several biological systems (Thaysen-Andersen and Packer, 2014; Alves et al., 2017). Exosomes were isolated from urine from three healthy individuals using differential centrifugation. After tryptic digestion, glycopeptides were enriched with size exclusion and SNA lectin chromatography before nanoLC-MS/MS analysis using CID fragmentation. The data were analyzed by the GlycopeptideID software. In total 126 N-glycopeptides belonging to 37 glycoproteins were identified (Saraswat et al., 2015). Interestingly, the common markers utilized to characterize the purity of an exosomal preparation are glycoproteins such as CD81, CD63, CD9, CD82, and HSC. Moreover, several members of the galectin family and glycan binding proteins were identified in EVs (Heijnen et al., 1999; Gonzalez-Begne et al., 2009; Looze et al., 2009).

A particular feature of protein glycosylation in EVs is the differential glycan composition compared to the parental cell membranes. This indicates an enrichment of specific glycosylated antigens in the EVs and the secretion of glyco-epitope to fulfill diverse biological functions.

#### Ubiquitylation, SUMOylation, and ISGylation in EVs

Ubiquitylation is the process involving the addition of ubiquitin, an 8.5 kDa protein, in the form of monomeric or polymeric moieties forming an isopeptide bond between the C-terminal end of ubiquitin and the lysine side chain of the substrate. Small Ubiquitin Modifier (SUMO) is a 12 kDa ubiquitin-like protein. Protein ubiquitylation and SUMOylation control several biological processes such as protein degradation, DNA damage, autophagy, protein sorting, among others (Komander and Rape, 2012). Comprehensive reviews on sample preparation and LC-MS analysis of protein ubiquitylation and SUMOylation have been reported elsewhere (Peng et al., 2003; Xu et al., 2009, 2010; Blomster et al., 2010; Impens et al., 2014; Tammsalu et al., 2014).

An initial study looked at the presence of ubiquitylated proteins in exosomes isolated from EBV-transformed human B-cell line and mouse immature splenic dendritic cells. Exosomes were isolated by differential centrifugation and exosomal membranes were stripped by sodium carbonate treatment. Using immunodetection with anti-ubiquitin (P4D1) and mouse anti-polyubiquitin (FK1) it was shown a majority of soluble polyubiquitinated exosomal proteins (Buschow et al., 2005). Proteomic screening of microvesicles isolated from plasma of healthy donors identified 161 proteins, such as ubiquitin, suggesting the presence of ubiquitinated proteins in exosomes as previously reported (Bastos-Amador et al., 2012). Even though this study did not report any direct evidence of protein ubiquitylation, it did reveal high variability of kinase and the protein content between individuals. On the other hand, protein ubiquitylation was identified in exosomes released from myeloid-derived suppressor cells. Exosomes were isolated through differential centrifugation and lysed in urea buffer before applying a GeLC-MS strategy. Ubiquitinated proteins were enriched at protein and peptide level using anti-ubiquitin and diglycine remnant antibodies, respectively. Tryptic peptides were analyzed by LTQ-Orbitrap mass spectrometry and fifty ubiquitinated proteins were identified at 5% FDR (Burke et al., 2014). Tryptic digestion of ubiquitinated proteins generate a diglycine remnant with a +114.00 Da mass difference which can be enriched with a specific antibody against this epitope. A recent investigation of exosomes isolated from six healthy donors identified 619 ubiquitinated proteins using a GeLC-MS approach on enriched ubiquitinated peptides. Interestingly the majority of the identified sites were novel compared to the ones reported in public repositories (Huebner et al., 2016). Moreover, SUMOylation of hnRNPA2B1 was shown to regulate the miRNA sorting into EVs (Villarroya-Beltri et al., 2013). More studies are needed to investigate the SUMOylation in EVs in different physiological conditions.

**Figure 4:**
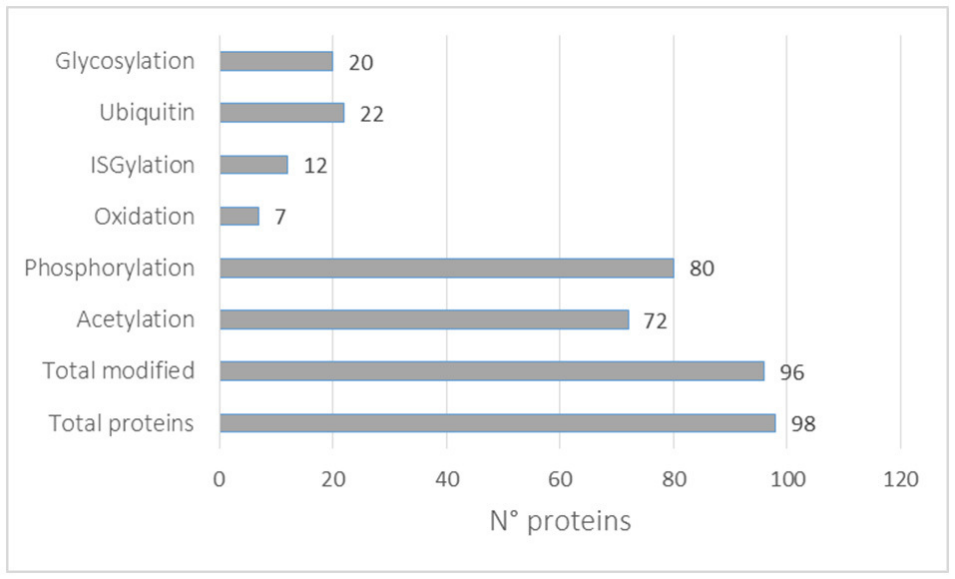
PTMs distribution in the top 100 exosomal proteins reported in the Exocarta database.

In this review, we looked at the reported PTMs of the top 100 proteins mostly identified in exosomes according to the Exocarta database. In total, 98 proteins were mapped to the Uniprot database and all the reported PTMs were assembled as shown in Supplementary Table 6. Almost all proteins were reported to be post-translationally modified (96) with the highest number of them being phosphorylated (80), acetylated (72), ubiquitinylated (22), and glycosylated (20). It should be noted that each protein can be modified with different PTMs. Moreover, the higher presence of some PTMs might be correlated with the availability of analytical methods, as described above. Interestingly, 12 of the 98 proteins mostly identified in exosomes were reported to be ISGylated. ISGylation post-translational modification involves the covalent addition of Interferon-stimulated gene 15 (ISG15) protein through an isopeptide bond similar to ubiquitin. ISG15 induction is primarily triggered by Type I IFNs and is conjugated to protein substrates through several enzymes. ISGylation is involved in innate immunity, antiviral/antibacterial response and cancer (Zhang and Zhang, 2011). Recently ISGylation was found to control exosome secretion and lysosomal degradation (Villarroya-Beltri et al., 2016). More studies are needed to identify ISGylated proteins in EVs. It should be noted that the majority of the PTMs reported in Figure 4 were not identified in EVs preparations and more studies are necessary to validate each modification in EVs and to catalog the EVs PTMome in different pathophysiological conditions.

PTMs do not act alone, but combinatorically regulate cell functions in an intricate cross-talk network (Melo-Braga et al., 2012; Palmisano et al., 2012b; Huang et al., 2016). One of the first studies that looked at PTM cross-talk in EVs was published by Palmisano et al. (2012a). In this study, the authors isolated by differential centrifugation exosome and microvesicles from a rat insulinoma cell line (NHI 6F Tu28). Exosome and microvesicles were derived from β-cells after cytokine induced apoptosis. EVs proteins were digested with trypsin and glycosylated and sialylated glycopeptides were enriched using titanium dioxide chromatography before LC-MS/MS analysis. A quantitative comparison was performed using stable isotope labeled amino acids in cell culture combined with mass spectrometry. A total of 401 and 191 proteins were identified in MPs and exosomes, respectively. In addition, 151 phosphorylation sites and 239 sialylated N-glycosylation sites on proteins originating from β-cell-derived microvesicles were reported. Interestingly the TNF receptor was up-regulated in microvesicles released from cytokine-stimulated insulinoma cell line.

The computational and functional analysis of multiple PTMs is still a challenge but elucidating their cross-talk will help in picturing the PTM scenery in EVs and offer novel biomarker and therapeutic targets (Zahedi, 2016). Association of bottom-up proteomics with diverse enrichment methods allows the identification of multiple PTM sites in EVs. Moreover, the combination of several omics techniques such as metabolomic, lipidomic, and transcriptomic will help in understanding the complex biological network of EVs (Coman et al., 2016; Quinn et al., 2016; Tisoncik-Go et al., 2016).

PTM analysis of EVs proteins in non-human species is lacking behind, maybe due to the lack of annotated protein databases, the presence of non-canonical PTMs and the quite recent discovery of their importance. Future studies need to apply proteogenomic strategies to deeply characterize the EV proteome. Thus, we stimulate the EVs research community to dig deeper in these protein modifications and apply them to different biological systems.

#### EVs as carriers of mutated and misfolded proteins

Additionally, exosomes have been involved in the extracellular transfer of misfolded or differentially modified proteins. Site-specific protein mutations alter the structure and function of proteins influencing their biological properties. For example missense mutations in KRAS, which lock the protein into the GTP-bound state, occur in 30–40% of colorectal cancers (Pylayeva-Gupta et al., 2011). In a recent study, KRAS was found secreted in exosomes, which were able to transfer mutant KRAS to recipient cells expressing only wild-type KRAS. This transfer increased the aggressiveness of wild-type KRAS cells (Demory Beckler et al., 2013). The transfer of EGFRvIII has been shown in glioblastoma and its functional activity has been demonstrated through a sustained activation of the signaling pathway. Mutated proteins were found in several exosomal preparations, showing the importance of EVs in preconditioning adjacent tissue for tumor growth. This field effect can be exerted locally or at the distant metastatic site (Chai and Brown, 2009). Extracellular vesicles have been involved in loading and secreting aggregation-prone proteins such as α-synuclein (Emmanouilidou et al., 2010), phosphorylated tau detected in exosomes from human CSF (Saman et al., 2012) abnormally folded prion protein scrapie (PrPsc) (Fevrier et al., 2004), and β-amyloid (Perez-Gonzalez et al., 2012) involved in neurodegenerative disease.

In a recent study the proteome of exosomes (30–100 nm in diameter) and ectosomes (100–1,000 nm in diameter), isolated from neuroblastoma cells using OptiPrep^TM^ density gradient centrifugation, were compared. Using a proteogenomic approach, which combined exome sequencing with proteomic analysis in an integrated bioinformatics pipeline, the authors revealed mutant/aberrant proteins secreted via EVs (Keerthikumar et al., 2015).

Another important aspect of applying proteomic strategies to EVs is the possibility to identify missing proteins in the human protein map. Indeed, looking at a subset of proteins encapsulated in vesicles could help in reducing the dynamic range commonly encountered in complex biological samples such as tissues and biofluids. Within the guidelines of the Chromosome-centric Human Proteome Project, Guo et al. reported the identification of 1,091 phosphosites in exosomes isolated from SW620 colorectal cancer cells. Several new phosphosites were identified and exosomal phosphoproteins were mainly located in the 11q12.1–13.5 region of chromosome 11 (Guo et al., 2016). The identification of low abundance proteins in EVs compared to the whole cell lysate suggests a specific and selective sorting of proteins into EVs and this could help in identifying proteins which are normally outside the dynamic range achievable by current instrumentation and methods and help filling in the gap of translated protein annotation.

### Top-down approaches and structural analyses to investigate EVs protein assembly and topology

Bottom-up proteomics approaches have allowed the identification and quantification of the complete proteome of biological systems (Yates et al., 2009; Aebersold and Mann, 2016). The bottom-up proteomic technologies have allowed the identification of thousands of proteins in EVs showing great promises for this technology (Pocsfalvi et al., 2016). However, bottom up approaches has some limitations: (1) protein inference issue due to the identification of peptides as protein surrogate, (2) sequence coverage, (3) underestimation of PTMs and sequence variations, and (4) loss of combinatorial PTM code. Top-down proteomics applies MS to sequence intact proteins and their proteoforms without the need of proteolytic digestion (Zhang and Ge, 2011; Toby et al., 2016). Several technological improvements in protein extraction, prefractionation, MS and fragmentation techniques were needed to achieve a proteome-scale top-down analysis (Tran et al., 2011; Toby et al., 2016). Top-down proteomics was applied for the study of low molecular weight (<39 kDa) proteins of exosomes isolated from murine myeloid-derived suppressor cells (Geis-Asteggiante et al., 2015). Total proteins and histones were extracted with 8M Urea and the EpiQuik Total Histone kit, respectively. Proteins were precipitated, solubilized in SDS and separated by GELFrEE. Fractionated proteins were analyzed by C3 reversed phase chromatography coupled to LTQ-Orbitrap XL MS. A total of 21 proteins with more than 200 proteoforms were identified. Forty-four proteoforms belonged to S100 protein family. Moreover, numerous histone variants with several PTMs were identified such as the proteolytical cleavage of 22 and 21 amino acid residues for histone H3.2 and H3.3, respectively (Geis-Asteggiante et al., 2015). This study shows the importance of top-down approaches in elucidating the great diversity of proteoforms. Despite the recent improvements for top-down and middle-down approaches, there still are technological challenges whose limit is widespread. More studies on exosomal proteins using top-down approaches will aid in characterizing specific proteoforms present in EVs compared to the parental cells.

Proteins do not act as single entity within a cell but build interaction networks which influence the phenotype (Vidal et al., 2011; Bensimon et al., 2012). Indeed, the phenotype of a biological system is the result of intracellular networks continuously rewired under external stimuli (Charbonnier et al., 2008). Protein-protein interaction (PPI) networks are crucial to understand the phenotype of a biological system. Mass spectrometry-based proteomics methods have been developed to study PPI and become the method of choice allowing large scale studies (Gavin et al., 2006; Krogan et al., 2006; Huttlin et al., 2015). Affinity-purification (AP) combined with mass spectrometry has been largely applied since it can detect PPI in physiological conditions such as cells and tissues (Zhou and Veenstra, 2007; Meyer and Selbach, 2015; Smits and Vermeulen, 2016). In this approach, the protein of interest (bait) is captured using specific antibodies or affinity probes against epitope tags for subsequent identification of its interactors (preys) (Gingras et al., 2007). A major challenge of AP-MS has been the distinction between true interactors and unspecific binders. This problem has been solved using tandem affinity purification strategy, in which the protein of interest is fused with two tags for sequential affinity purification (Puig et al., 2001). Due to the high sensitivity of current mass spectrometers, unspecific binders are detected and quantitative AP-MS methods were developed (Trinkle-Mulcahy, 2012; Baymaz et al., 2014). The label-based or label-free quantitative AP-MS allows the quantification of interacting proteins which co-isolate with the bait or a negative control. True interactors will have specific detection while unspecific binders will have a 1:1 ratio (Choi et al., 2010; Hubner et al., 2010). Although, very powerful, AP-MS suffers from using an overexpressed and/or tagged protein within the cell. This issue has been overcome using antibodies against endogenous proteins or silencing the protein of interest (Selbach and Mann, 2006). Alternatively, nanobodies can be used with higher affinity constant and specificity (Shi et al., 2015). Antibody-free methods have been developed using promiscuous biotin ligase fusion protein (Roux et al., 2012) and ascorbate peroxidase labeling (Chen et al., 2015). Alternatively, size exclusion chromatography or ion exchange chromatography have allowed the identification of hundreds of complexes in less time (Wan et al., 2015). Despite the detection of interacting proteins, an important question is the topology of the complex. Crosslinking freezes the protein structure and its interaction partners through covalent bond allowing the detection of transient and *in vivo* interactions (Rappsilber and Mann, 2007). Recently a proteome-wide crosslink approach was applied (Liu et al., 2015). Other techniques such as hydrogen-deuterium exchange (Rand et al., 2014) and limited proteolysis have been used to study protein complex topologies (Feng et al., 2014). Cryo-electron microscopy is a complementary technique to MS to study protein complexes (Alber et al., 2007). Cryo-electron microscopy was used to study the interaction of protein complexes EPCAM-CLDN7 and TNIK-RAP2A in human primary colorectal cancer cell exosomes (Ji et al., 2013). Choi et al. interrogated proteomic data obtained from EVs isolated from human colorectal cancer cells in order to create an interaction map (Choi et al., 2012) and some of these interactions were validated. Moreover, it allowed the identification of the Src complex for the EVs biogenesis. These studies suggest that the technology is ready to be applied to study the EVs interactome and to understand several processes related to protein-protein interaction.

**Figure 5:**
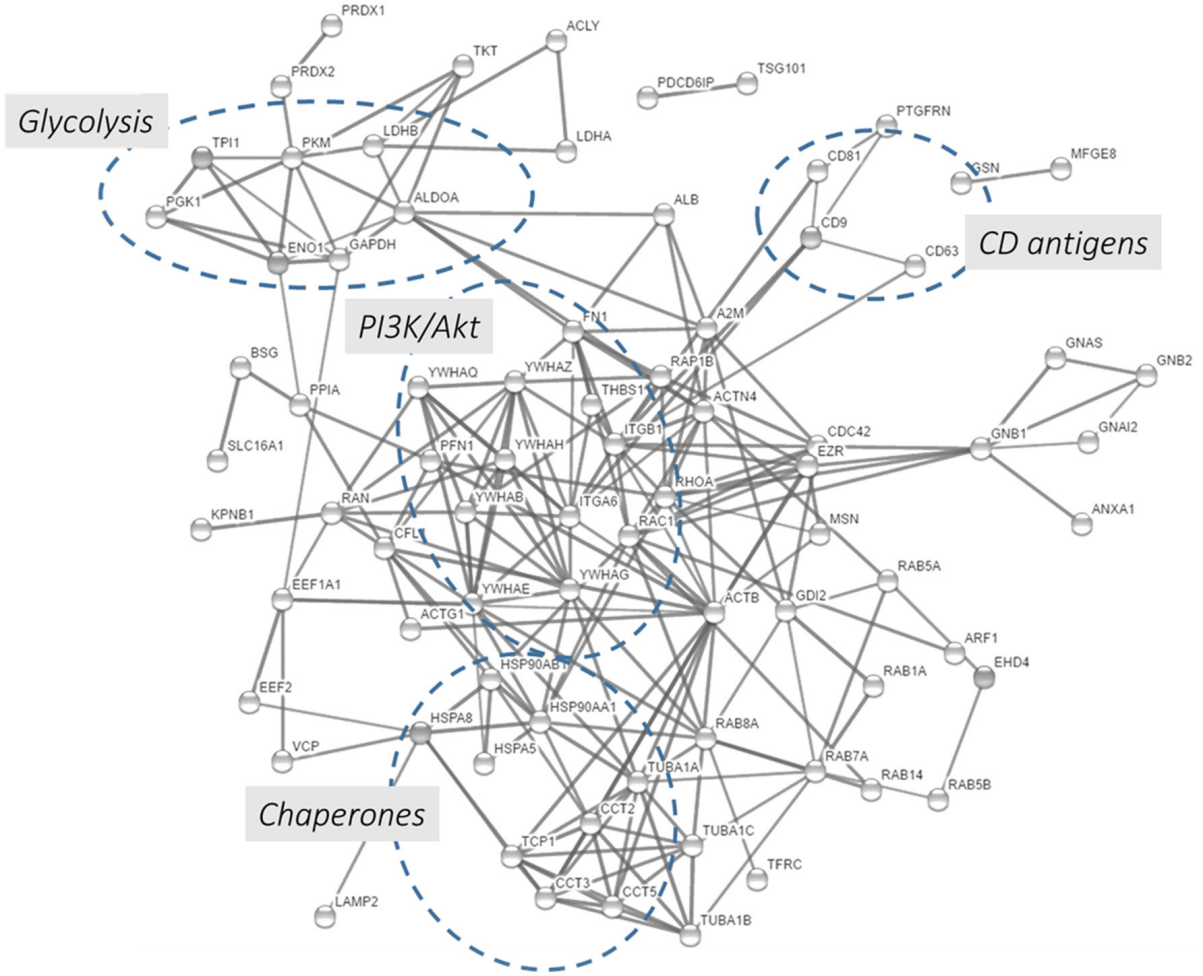
Protein-protein interaction network using the 100 proteins mostly detected in exosome studies. The 100 proteins mostly identified in the exosomes were downloaded from the Exocarta database and analyzed by the String database (http://string-db.org/; Szklarczyk et al., 2015). Direct protein-protein interactions were selected with 0.7 confidence without the text-mining function was selected for creating the network.

In this chapter, we performed a protein-protein interaction network analysis using the top 100 proteins identified in exosomes preparations according to the Exocarta database. Using the String functional annotation protein interaction database, we were able to build a protein-protein interaction network showed in Figure 5 and Supplementary Table 7. Ninety-seven nodes with 219 edges were identified and connected in this network with a statistical significance. Several hubs were identified such as proteins involved in the glycolysis, PI3K/Akt pathway, CD antigens, and chaperones. The presence of metabolic and cell signaling pathways highlights the importance of exosomes in different biological processes. The presence of few hubs in the interaction network and the enrichment of few signaling and metabolic networks are related to the size of protein list, in this case 100 proteins were loaded into the STRING database. Due to that, the enrichment of other pathways/networks would be possible by using a more comprehensive list of exosomal proteins.

It should be noted that the PPI analysis presented here along with the previously cited study (Choi et al., 2012) are based on post-acquisition data mining. In this review, the list of top 100 identified EVs proteins, deposited in the public repositories, were subjected to gene ontology enrichment tools and PPI interaction databases. These analyses correlated the current finding with previous knowledge about PPI, visualizing their interactions and the enriched biological processes. Although, these approaches are valuable to get a first hint into the enriched biological processes, more data are needed to confirm these interaction in EVs and study their perturbation in different pathophysiological conditions. Due to that, the research community working with EVs should apply MS-based and complementary (e.g., cryo-EM) techniques to identify the integrative networks presents in EVs looking with systems biology eyes and begin the era of EVs protein-interactome.

Although, exosome biogenesis is still under investigation, it has been shown that topology of surface proteins in exosomes is the same as plasma cell membranes from which they are derived. The same topology has been observed in microvesicles in contrast to endosomes, which show inverse topology (Ostrowski et al., 2010). The orientation of proteins in exosomes is crucial to modulate their interaction with recipient cells and modulate biological processes such as cellular signaling and immune response. Indeed, the molecular chaperone Hsp70 is located on the membrane of exosomes released from macrophages after mycobacterial infection or heat-stress. The topology of Hsp70 on exosomes allows the interaction with TLRs of recipient cells activating the NFKβ signaling pathway (Anand et al., 2010). Galectin 5 and 9 have also been identified on exosomal membranes highlighting their function in exosome uptake and immune modulation (Barrès et al., 2010). These studies focused on single protein and a detailed characterization of the topology of the EVs proteome was missing. In a recent report, the protein topology in EVs was assigned using two proteomic approaches based on limited proteinase-K (PK) treatment and biotin labeling of surface proteins (Cvjetkovic et al., 2016). In particular, EVs were isolated from human mast cells HMC-1 using differential centrifugation combined with a discontinuous iodixanol gradient. Initially the EVs were treated with proteinase-k which cleaves the surface exposed portions of proteins and do not penetrate the EVs leaving intact the luminal proteins. As a proof of principle, CD81, a common exosomal marker, was cleaved by PK due to its membrane localization. Subsequently, a quantitative large scale proteomic comparison between PK-treated and untreated EVs was carried out and 758 PK-sensitive proteins were identified. Five hundred and seventy of PK-sensitive proteins were annotated as non-membrane proteins. In parallel, biotin labeling of tryptic digested surface proteins was performed. The biotinylated peptides were enriched and analyzed by nLC-MS/MS. One hundred and fifty-five proteins were identified with 99 being annotated as non-membrane proteins. The overlap between the two methods revealed 14 proteins. In addition, 410 PK-sensitive membrane proteins were analyzed using the protein topology visualization tool Protter (http://wlab.ethz.ch/protter/). Based on a scoring system which compared the experimental data with the topology information, proteins were classified in conventional topology, inside-out and inconclusive. Comparing the two experimental methodologies, 4 proteins were overlapping and classified as inside-out meaning these proteins had an inverse topology in EVs compared to their cellular one. The inside-out topology was confirmed for the STX4 and SCAMP3 proteins by western blotting and flow cytometry (Cvjetkovic et al., 2016). The importance of elucidating the 3D structure of proteins and structural changes of exosomal proteins can reveal novel therapeutic targets for diseases.

## Conclusions and future directions

This review highlights the importance of proteomic strategies on the EVs protein characterization. Nowadays, high accuracy, sensitive and robust bottom-up proteomic technologies have boosted our understanding of the EVs protein content. However, the field has several challenges needed to be addressed in the future, as reported in Table 1. It will be imperative to better characterize the EVs subpopulations and their biomolecular markers (Kowal et al., 2016). Faster, more reproducible, high-throughput, and highly accurate relative and absolute protein quantification methods need to be routinely applied to the EVs field (Egertson et al., 2013). There is a need for improving the sequence coverage of the EVs proteome to identify their PTMs, mutations, and proteoforms (Keerthikumar et al., 2015). The protein-protein interaction network and protein structure will be an additional level of information for better biomarkers and therapeutics (Choi et al., 2012). Proteomic combined with other omics sciences such as lipidomic, metabolomic, and transcriptomic will give a systems biology overview on EVs (Coman et al., 2016). Moreover, the application of proteomic approaches to EVs isolated from tissues (Perez-Gonzalez et al., 2012) and the assignment of biological functions to EVs proteins within a pathophysiological context will allow provide a translational perspective to future studies (Iraci et al., 2016). A biology-oriented view on EVs proteins is needed to further explore their potential as theranostic tools.

**Table 1:** Challenges and future directions in EVs proteomics.

**Challenges**	**Possible solutions**	**References**
1. Isolation of homogeneous EVs population.	Optimize and standardize protocols for EVs isolation.	Kowal et al., 2016
2. Accurate, robust and high-throughput quantification of protein and PTMs in EVs.	Data-independent and Targeted proteomics.	Egertson et al., 2013
3. Improve the EVs proteome sequence coverage.	Using multiple proteolytic enzymes, fractionation techniques, and LC-MS strategies.	Swaney et al., 2010
4. Identify the PTMome (the post-translational protein complement) of EVs.	The application of existing and development of novel enrichment methods for PTMs.	Palmisano et al., 2012a
5. Identify mutated proteins in EVs.	Proteogenomics strategies combining RNAseq data with proteomic data.	Keerthikumar et al., 2015
6. Map protein-protein interaction networks in EVs.	Applying mass spectrometry-based protein-protein interaction methods such as AP-MS, XL-MS, and native MS.	Choi et al., 2012
7. Identify EVs proteoforms and combinatorial PTMs.	Top-down and middle-down proteomic approaches could allow a deeper identification of the combinatorial PTMs.	Geis-Asteggiante et al., 2015
8. Integrate multi-omics strategies to unravel the systems biology makeup of EVs	Perform multi-omics strategies to identify and quantify different biomolecules with the aim of looking at the complete biological picture.	Coman et al., 2016
9. Characterize the proteome of EVs isolated from primary cell lines and tissues.	Apply high accuracy and sensitive MS to minute amount of sample and develop novel extraction methods for tissues-derived EVs.	Perez-Gonzalez et al., 2012
10. Functional validation of EVs secreted proteins on the biological state of recipient cells.	Apply cell biology, genomics, *in vivo*, and translational techniques to understand the role of EVs.	Iraci et al., 2016

## Author contributions

LR-F and GP designed the review and wrote the review. VR, VC, and AU revised the review. GP conducted and systematized the review. All authors have read and agreed with the final version of the review.

### Conflict of interest statement

The authors declare that the research was conducted in the absence of any commercial or financial relationships that could be construed as a potential conflict of interest.

## Supplementary material

Supplementary Table 1 Top 100 proteins expressed in the Exocarta along with their gene ontology information.

Supplementary Table 2 Top 100 proteins expressed in the EVpedia database.

Supplementary Table 3 Proteins detected in the Extracellular vesicles of the Plasma Proteome Database.

Supplementary Table 4 Gene Ontology Analysis of the top 100 proteins identified in the exosomes according to the Exocarta database.

Supplementary Table 5 EVs protein markers.

Supplementary Table 6 Distribution of PTMs in the top 100 proteins identified in exosomes preparations and reported in the Exocarta database.

Supplementary Table 7 KEGG pathways (http://www.genome.jp/kegg/) enriched in the dataset of the top 100 proteins identified in exosomes preparations according to the Exocarta database.
